# Citrus Allergy from Pollen to Clinical Symptoms

**DOI:** 10.1371/journal.pone.0053680

**Published:** 2013-01-04

**Authors:** Rosa Anna Iorio, Stefano Del Duca, Elisabetta Calamelli, Chiara Pula, Magda Lodolini, Fortuna Scamardella, Andrea Pession, Giampaolo Ricci

**Affiliations:** 1 Department of Biological, Earth and Environmental Sciences, University of Bologna, Bologna, Italy; 2 Department of Pediatric Allergology of the Women's, Children's and Adolescents' Health Gozzadini Children's Hospital Sant'Orsola-Malpighi, University of Bologna, Bologna, Italy; 3 Laboratory Analysis Unit. Maggiore Hospital, Bologna, Italy; Murdoch University, Australia

## Abstract

Allergy to citrus fruits is often associated with pollinosis and sensitization to other plants due to a phenomenon of cross-reactivity. The aims of the present study were to highlight the cross-reactivity among citrus and the major allergenic pollens/fruits, throughout clinical and molecular investigations, and to evaluate the sensitization frequency to citrus fruits in a population of children and adults with pollinosis. We found a relevant percentage of sensitisation (39%) to citrus fruits in the patients recruited and in all of them the IgE-mediated mechanism has been confirmed by the positive response to the prick-to-prick test. RT-PCR experiments showed the expression of Cit s 1, Cit s 3 and a profilin isoform, already described in apple, also in *Citrus clementine* pollen. Data of multiple sequence alignments demonstrated that *Citrus* allergens shared high percentage identity values with other clinically relevant species (i.e. *Triticum aestivum, Malus domestica*), confirming the possible cross-allergenicity citrus/grasses and citrus/apple. Finally, a novelty of the present work has been the expression of two phospholipaseA2 isoforms (PLA2 α and β) in *Citrus* as well as in *Triticum* pollens; being PLA2 able to generate pro-inflammatory factors, this enzyme could participate in the activation of the allergenic inflammatory cascade.

## Introduction

The regular consumption of fruit and vegetables is generally encouraged also by the European Community as the basis of a daily diet healthy and able to improve human health and prevent some diseases: there are many data that confirm the direct correlation between the consumption of fresh fruit and the reduction in the incidence of cardio-vascular disease, asthma, diabetes and cancer [1], [2]. The benefit derives not only from their nutritional properties in terms of vitamins, minerals and fibres, but also from the “non-nutritional” component, such as the high content in secondary metabolites (phenols, flavonoids, carotenoids) that play an important antioxidant role.

Unfortunately, about 1/5 of the population in Western countries suffer from respiratory allergies [3]. Recent epidemiological studies estimate that food allergies are increasing and affecting millions of people (1–2% of world population), as to consider them as the epidemic of the XXI century [4]. Nearly 4% of the US populations are afflicted with food allergies, a prevalence much higher than appreciated in the past. In addition, the frequency of food hypersensitivities is greater in the first few years of life, affecting about 6% of infants less than 3 years of age and decreasing over the first decade [5].

For food allergies there is still no effective treatment and, consequently, up to now the only therapy is still the avoidance of fruits that cause allergy [6] and, considering that a regular consumption of fruits is reported to enhance human health and prevent various diseases, it is easy to understand that allergy can significantly affect the quality of life of allergic patients in a profoundly negative way [7]. The problem is compounded by the fact that food allergy may also arise as a result of cross-pollen allergy [8].

Oranges (*Citrus sinensis*) are largely consumed worldwide and commonly included in the population's diet in many countries, both as fresh fruit or derived beverages (juices) and foods (jams) [9], [10]. This fact has probably led to point to orange among the main allergenic plant food in a public perception survey of food allergies [11]. Although in the daily clinical practice orange allergy is obviously rarely observed in Central Europe, some early studies suggested oranges being an important allergenic food, as for other foods and pollens such as kiwi, strawberry, peach, banana, grasses (www.foodallergyitalia.org) [12]. However, allergy to oranges or other citrus fruits has been scarcely investigated [10]; when present, it is often associated with pollinosis and sensitization to other plants [10] due to a phenomenon of cross-reactivity, whereby the pollen would be the cause of a sensitization by the respiratory way that could predispose to allergy towards foods that contain homologous proteins to those in sensitizing pollen. In an English study on 67 asthmatic children which compared a multiple food specific IgE antibodies (sIgE) test to parental perception of food allergy, oranges resulted implicated in the 15% of patients [13]. Another study reported a frequency of 17% of adverse food reactions after eating oranges in a population of 100 adults suffering from oral allergy syndrome (OAS) as common manifestations of allergy to oranges [14]. Previous studies indicated mild local reactions and described subjects affected from orange-dependent exercise-induced anaphylaxis after eating oranges [12].

The three major orange allergens that have been recently identified and characterized are Cit s 1, a germin-like protein (GLP) [14], [15], Cit s 2, a profilin [9], [10] and Cit s 3, a new member of the lipid transfer protein pan-allergen family (LTP) [9]. Cit s 1 is a glycoprotein of 24 kDa recognized by patients' sera IgE recently described by Ibáñez et al. [15] and Pignataro et al. in lemon fruit flavedo [16]; its biological activity in fruit need to be clarified, but it is considered one of the major orange allergen [12] displaying an high *in vitro* reactivity with its glycans constituting the major IgE epitopes [9]. Moreover, the protein could be present in different isoforms ranging from 20 to 120 kDa [16]. Cit s 2 represents the other major allergen according to its *in vitro* and *in vivo* reactivity in patients with allergy to this fruit [10]. Its biological activity is associated to the structural organization of actin filament; in particular it is believed to be involved in the transition between G- and F-actin, playing a very important role for pollen germination and indeed for plant fertilization [17]; its reactivity with patients'sera is associated to plan pollen allergy [18]. Cit s 3 behaves as a minor allergen (approximately 35% prevalence) [9], contrary to the fact that LTPs are considered in general the major fruit allergen in the Mediterranean area [12]. Cit s 3 is present in pulp, but in lower amount comparing to flavedo, so the moderate IgE reactivity observed might be explained by the low nsLTP concentration in the orange pulp [19]. LTP role in plant is believed to be related to the defence mechanisms and in the control of pathogen attack responses. Moreover, a role in transporting lipid molecules to the outer layers of plant organs, in embryogenesis and in abiotic stress response has been also proposed [20]. The multiple function of LTPs is also supported by the presence of isoforms showing moderate levels of amino acid sequence identity and different gene expression patterns [21].

Ahrazem et al. [19] sought to identify and isolated citrus fruit LTPs and to explore their relevance in orange allergy in 27 patients with OAS after orange ingestion and with positive skin-prick test (SPT) as well as prick-to-prick test (PPT) responses and sIgE levels to orange. This study showed that members of the LTP allergen family are involved in orange's allergy, displaying positive *in vivo* and *in vitro* tests in 30–50% of the subjects studied. Furthermore, both orange and lemon allergens showed cross-reactivity with the major peach LTP allergen Pru p 3: the recombinant orange isoform (rCit s 3) presented 67% sequence identity with rPru p 3 [19].

Crespo et al. [12] documented a statistically significant correlation between the presence of IgE mediated sensitization to the two orange major allergens, Cit s 1 and Cit s 2 and the positive response to Skin-Prick test (SPT) respectively to olive and cypress (Cit s 1) and to platanus (Cit s 2) in 56 patients with self-reported adverse reactions to oranges.

The purposes of the present study were to i) deeply investigate the cross-reactivity among citrus and the major allergenic pollens and fruits, either under clinical and molecular points of view, and to ii) evaluate the frequency of sensitization and/or allergy to citrus fruits (oranges, lemons and clementines) in a population of children and young adults with pollinosis (allergic hay fever and/or asthma) recruited for this monocentric observational study.

The cross-reactivity aspect has focused on the analysis of i) the sIgE levels in patients serum in order to determine the major citrus allergens levels by the use of the recombinant molecules Pru p 3 (LTP protein homolog to Cit s 3), r Phl p 12 (profilin homolog to Cit s 2) and MuxF3-CCD, marker of sensitization for Cross-reactive carbohydrate determinants (CCD) and of ii) allergens expression (Cit s 1, Cit s 2, Cit s 3) in *Citrus clementine* pollen, in order to assess its potential allergenicity and cross-reactivity with other pollens, especially *Triticum aestivum*, whose allergens sequences are conserved in the *poaceae* family. The three important citrus allergens reported above were also analyzed in citrus pollen because of the possible cross-reactivity between pollen and fruits, as documented for apple [22]. *Citrus clementine* pollen can be potentially important from the allergenic point of view because present in some foods as marmalade and honey and for farmers cultivating citrus fields.

The research for sensitizing agents in pollen continued with the evaluation of the expression of two isoforms of secretory phospholipase (PLA2 α and β), key enzymes in the synthesis of pro-inflammatory eicosanoids that may have a key role in raising sensitization and allergic reaction [23], [24].

## Materials and Methods

### Chemicals and Antibodies

All chemicals (unless otherwise indicated) were obtained from Sigma-Aldrich (Milan, Italy).

### Plant material

Mature pollen of clementine (*Citrus clementine* Hort. Ex Tan. cv. Comune) was collected from flowers kindly donated by Professor S. Mazzucca, University of Calabria (Dipartimento di Ecologia), and pollen of *Triticum aestivum* (wheat) was kindly donated by Azienda Agricola Rondinini (Faenza, Ra). Handling and storage were performed as reported by Bagni et al. [25], stored at −20°C with NaOH pellets to maintain it dry.

Fruits of oranges, clementines and lemons have been provided by Azienda Agricola “San Mauro” di Minisci Edmondo (Corigliano Calabro, Cosenza) within the project “My Darling Clementine”: un prodotto salutistico nuovo e innovativo dalle clementine e dal limone di Calabria” (POR FESR 2007–2013).

### *In vitro* germination

Pollen was hydrated at 20°C-100% rHu for 1 h and allowed to germinate (1 mg/mL in germination medium: 15% sucrose, 324 µM boric acid, 2% agar) 13 h (overnight, o/n) into glass Petri dishes at 20°C-100% rHu.

### Total RNA isolation and cDNA synthesis

Total RNA was extracted from 30 mg of frozen ungerminated (UGP) and germinated (GP) *Citrus c*. and *Triticum a*. pollens following the basic protocol for phenol/SDS method [26] with minor modifications as published by Paris et al. [27]. The purity and concentration of the extracted RNAs were evaluated by measuring the absorbance at 260 and 280 nm wavelengths using a NanoDrop Spectrophotometer ND-1000 (EuroClone S.p.A., Italy). RNA was considered pure if the A260/280 ratio was grater than 1.8 and A260/230 ratio higher than 2. First-strand cDNA was synthesized following the instruction from Stratagene (Agilent Technologies, USA), starting from 1 μg DNA-free RNA, and 2 μl was used for the semi-quantitative PCR analyses.

### Primer design

Specific primer pairs (Table 1 and Table 2) were designed on the basis of the sequences obtained from National Center for Biotechnology Information (NCBI) Reference Sequence database (http://www.ncbi.nlm.nih.gov) with the software Primer3 version 0.4.0 (http://frodo.wi.mit.edu/primer3) and further tested with the software PrimerSelect® v8.0-MegAlign for the formation of primer homo and heterodimers. As endogenous control the EF1-α gene of *Citrus sinensis* (GenBank ID AY498567) and the UBQ10 (GenBank ID CB322134.1) genes were used.

**Table 1:** Primer sequences and amplicon characteristics for *Citrus sinensis* genes used in semi-quantitative and qRT-PCR.

Gene	GeneBank accension number	Sequence 5′–3′	Product size (bp)	Ta (°C)	qRT-PCR Efficiency *	R^2^ *
*UB10*	CB322134.1	GATCCCACCAGACCAGCAA	105	60	1,953	1
		ACCAAATGAAGGGTTGATTCCTT				
*EF1*	AY498567.1	ATTGACAAGCGTGTGATTGAGC	132	60	1,948	0,999
		TCCACAAGGCAATATCAATGGTA				
*Cit s 1*	UniProt P84159	CATTCCAGTTGGACCCAAAG	191	60	1,961	1
		CAGTCAGCCTGGAGAGAGGT				
*Cit s 2*	AJ865015.1	CTTTCCTGCGTTTAGGCTTG	189	60	n.d.	n.d.
		AGGGCCTGATTGGTCTTCTT				
*Cit s 3*	AJ783335.1	CCCTATACCTGTGCCATGCT	199	60	1,902	1
		GCAGTCAGTGGAGATGCTGA				
*PLA2* α	GU075396	GCCATAGCCCATGTTTTCAT	184	60	1,89	1
		CACTGTACAGCAGCCCACAG				
*PLA2 β*	GU075398	GTATCTCTGCAAGCGCACTG	180	60	1,961	1
		TGCCATGTCCATACCCTGTA				

* The qRT-PCR efficiency and correlations; R^2^ were determined with LinRegPCR software.

**Table 2:** Primer sequences and amplicon characteristics of *Triticum aestivum* (wheat) and *Malus domestica* (apple) cross-allergens.

Gene	GeneBank accension number	Sequence 5′–3′	Product size (bp)	Ta (°C)
*Tri a 12*	X89827.1	CCTTGCACCAACTGGTCTTT	162	60
		CCAGGAGTCATGGGTTCATC		
*Tri a 14*	AJ784902.1	GGCGTCAAGAACCTCCATAA	177	60
		GCTGCAGTCGATGTTGAGAC		
*Mal d 3.02*	AY572532.1	TGGCCAGGTGAGCTCCAA	247	60
		TGGTGGAGGTGCTGATCTTG		
*Mal d 4.02*	AY792613.1	GTGTTACTTGTCAAGAAGAGCACAA	126	58
		GCTCAATGAGATAATCCGCA		
*PLA 2.23*	EEF48118.1	TGGGAAGTATTGTGGGCTTT	259	52
		AGCAACTAAAGCAGCCTCCA		

### Allergen gene expression analysis by semi-quantitative PCR

The PCR reactions were performed in a 15 μl reaction containing 2 μl cDNA, 0.1 μM specific primers (Table 1 and Table 2), 1.5 mM MgCl2, 100 μM dNTPs, 0.5 Unit Taq DNA Polymerase (Fermentas, M-Medical, Italy) and 1X reaction buffer. The reaction included an initial 5 min denaturation step at 94°C, followed by 40 PCR cycles at 94°C 30 s, the optimised annealing temperature for 45 s and 1 min at 72°C.

The number of amplification cycles was chosen to be in the exponential phase of amplification. The amplicons were visualised with a transilluminator (Vilber Lournet, Genenco) at 302 nm after electrophoresis in 2% (w/v) agarose gels in TAE 1X Buffer, containing 0.5 μg/ml ethidium bromide, and photographed (Nikon E5400 Coolpix). The experiment was repeated twice.

### Real-time PCR (qRT-PCR) analysis

The transcript levels were determined by real-time quantitative RT-PCR (qRT-PCR) using a StepOnePlus^TM^ Real Time PCR System (Applied Biosystems, USA) and the Power SYBR Green PCR Master Mix (Applied Biosystems, USA) as recommended by the manufacturer. PCR reactions were carried out in 96-well plates (10 µl per well) in a buffer containing SYBR Green (including Taq polymerase, dNTPs, SYBR Green dye) and 200 nM of each primer (forward and reverse, Table 1). After denaturing at 95°C for 10 min, a two-step amplification occurred: 15 s of denaturation at 95°C and 1 min of annealing/extension at 60°C, with a total of 40 cycles. The melting curves were analyzed at 60°C-95°C after 40 cycles to ensure that the resulting fluorescence originated from a single PCR product did not represent primer-dimers formed during the PCR or a nonspecific product. Each qRT-PCR analysis was performed in triplicate. Negative controls without cDNA were routinely included.

qRT-PCR results were analyzed with the sequence detection software SDS version 1.1 (Applied Biosystems, USA). The SYBR green fluorescent signal was standardized to a passive reference dye (ROX) included in the PCR master mix. Direct detection of the PCR product was measured by monitoring the increase in fluorescence caused by the binding of SYBR green dye to double-stranded DNA.

To estimate the expression variation levels of the eight candidate genes, geNorm software package for Microsoft Excel (http://medgen.ugent.be/jvdesomp/genorm/) [28] was applied to analyze the data obtained from the samples of citrus pollen [29]. The PCR efficiency was calculated for each gene with LinRegPCR program [30] from raw fluorescence data taken from the Applied Biosystems detection system. Results from the LinRegPCR software were imported into Microsoft Excel, transformed to relative quantities using the comparative Ct (ΔΔCt) method, and taken into account the different amplification efficiencies for the different genes (just replace value 2 with the actual efficiency of the gene (e.g. 1.95 for 95%) in the formula of ΔCt [31]. Data have been normalized to the geometric mean of two housekeeping genes (EF-1 and UBQ 10), chosen according to the geNorm software.

### Multiple sequence alignments

The multiple sequence alignments of the sensitizing factors annotated in the UniProtKB database (http://www.ebi.ac.uk/uniprot/) has been performed through ClustalΩ tool (http://www.ebi.ac.uk/Tools/msa/clustalo/) to find sequence homology among *Citrus sinensis* and *Triticum aestivum, Phleum pratense, Cynodon dactylon, Betula pendula, Artemisia vulgaris, Parietaria judaica, Malus domestica, Corylus avellana, Olea europaea, Arachis hypogaea*.

### Study population for clinical trials

This monocentric observational study enrolled all caucasian children and young adults affected from pollinosis [allergic rhino-conjunctivitis (RC) and/or asthma] consecutively referred to the Allergologic Center of the Paediatric Department of Bologna University (Northern Italy) from December 2011 to May 2012.

The criteria for study inclusion were:

- aged between 4 and 22 years;
- diagnosis of allergic RC and/or asthma with or without sensitization/allergy to oranges;
- Written informed consent from all patients (or from parents for patients younger than 18 years old).

The criteria for study exclusion were:

- Children with height or weight less than 3^rd^ centile;
- Patient affected from systemic disease (different from allergy or asthma) including: gastro-esophageal reflux disease under medical treatment, epilepsy, severe neurological or neurodevelopmental disorders, tuberculosis, previous thoracic surgery, major congenital malformations, heart diseases (with the exception of atrial sept defect without hemodynamic significance and ventricular sept defect), primary or secondary immunodeficiencies.

### Allergometric assays

In each patient, the presence of symptoms of food allergy and/or OAS to oranges and other citrus fruits was investigated. Skin-Prick test (SPT) with commercial extracts (Lofarma, Milano) of pollens (grass, composites, parietaria) were performed as well as prick-by-prick test (PPT) with fresh fruit pulp of oranges, lemons and clementines. A SPT result was considered positive when elicited a wheal at least greater than half of histamine control.

In the patients with a positive PPT with citrus fruits pulp, the levels of specific IgE were determined to a panel of the main pollens (*Phleum p., Parietaria j., Olea e., Betula v., Corylus a*.), recombinant allergens (rPhl p 1, rPhl p 12, rBet v 2, rPru p 3), fruit extracts (orange, apple, peanut, wheat) and MuxF3-CCD, a marker of sensitization for cross-reactive carbohydrate determinants (CCD) (ImmunoCAP 1000 FEIA, ThermoFisher-Sweden). In addition, the choice of determining the sIgE levels for rPhl p 12 and rPru p 3 has been made on the base of the high degree of homology with orange profilin Cit s 2 (almost 75%) [32] and orange LTP Cit s 3 (almost 67%) [18] respectively. Levels of sIgE greater than 0.35 kU/L were considered positive.

### Data and Statistics

The values reported are expressed as mean ± SD; they have been calculated on the basis of the data obtained from three independent experiments run separately and each sample was undertaken in triplicate (total nine replicates for each determination). Differences between sample sets were determined by the Student's *t*-test with 95% confidence limits. When indicated statistical analysis was performed using GraphPad Prism (version 5.03 Windows GraphPad Software Inc., La Jolla, CA, USA).

### Ethic Statement

The research was conducted according to the principles expressed in the Declaration of Helsinki, and approved by Ethic Committee of Bologna University, S. Orsola-Malpighi Hospital (protocol name: OrangeAllergy; number: 108/2011/U/Oss.). Written informed consent was obtained from all the parents or guardians of the minors involved in the study.

## Results

### Clinical results

We have recruited 72 children and young adults (50 males, 69% and 22 females, 31%) with mean age of 12,3 years (range 6–22 years) and clinical history of pollinosis, who presented a positive reactions to SPT with grass pollen extract.

Forty-seven patients (65%) had symptoms of allergic RC, 25 (35%) presented both RC and asthma. Nine patients (12%) referred systemic symptoms after ingesting the following foods (alone or in combination): 6 to nuts (2 to hazelnut, 1 to peanut, 1 to walnut, and 2 to a not specified nut), 2 to citrus fruits (1 to orange and 1 to clementine), 1 to kiwi, 1 to apple, 1 to tomato, 1 to egg, 1 to fish and 1 to milk. Four patients (6%) presented symptoms of OAS to the following plant-derived foods (alone or in combination): 2 to kiwi, 2 to melon, 1 to orange, 1 to strawberry and 1 to peanut. Overall three out of patients (4%) showed symptoms after ingestion of citrus fruits (2 systemic food allergy – 1 to orange and 1 to clementine – and 1 OAS to orange). The patient with systemic orange allergy showed a severe reaction with urticaria and vomiting immediately after ingestion of orange, otherwise the other patient with systemic citrus allergy presented abdominal pain and diarrhea after the assumption of a clementine. The case of OAS due to citrus fruits was elicited by the contact with an orange. The clinical features and allergological assays of the three patients with documented citrus fruits allergy are reported in Table 3.

**Table 3:** Clinical features of the three patients with citrus fruit allergy.

**PATIENT DATA**	Patient	**1**	**2**	**3**
	*Sex*	F	M	M
	*Age yrs*	20	20	12
	*Respiratory disease (A/RC)*	A, RC	A, RC	RC
	*Symptoms with citrus fruits*	Urticaria Vomiting (Orange)	OAS (Orange)	Abdominal pain Diarrhoea (clementine)
	*Food Allergy/ OAS to other foods*	apple, nuts, tomato	kiwy, melon, strawberry	kiwy
**PRICK-TO-PRICK WITH FRUIT PULP ** *Mean wheal diameter* **** *(mm)*	*Orange*	7	4	0
	*Clementine*	6	3	3
	*Lemon*	6	0	0
**SPECIFIC IGE (KU/L)**	*Orange*	39.2	<0.10	4.3
	*Wheat*	14.3	<0.10	6.5
	*Apple*	45.7	<0.10	24.4
	*Peanut*	47.8	<0.10	13.1
	*Phleum p.*	87.8	14.3	83.9
	*Olea e.*	56.8	0.8	33.3
	*Betula v.*	>100	<0.10	57.3
	*Corylus a.*	92.7	0.4	59
	*r Phl p 1*	41	15.1	71.2
	*r Phl p 12*	34.2	0.31	4
	*r Bet v 2*	47	<0.10	7.1
	*r Pru p 3*	19.7	<0.10	16.6
	*MuxF3-CCD*	1.23	<0.10	0.74

Patient data, results of prick-to-prick (PPT) with fresh citrus fruit pulp and specific IgE (sIgE) against the main pollens, (*Phleum p., Olea e., Betula v., Corylus a*.), fruit extracts (orange, apple, peanut, wheat), recombinant allergens (rPhl p 1, rPhl p 12, rBet v 2, rPru p 3) and MuxF3-CCD.

A: Asthma; OAS: oral allergy syndrome; RC: rhino-conjunctivitis; yrs: years.

PPT with the pulp of fresh orange, lemon and clementine resulted positive in 28 patients (39%): 24 (33%) to orange (mean wheal diameter  = 2.8 mm, range: 2–7 mm), 16 (22%) to lemon (mean wheal diameter  = 2.8 mm, range: 2–6 mm) and 12 (17%) to clementine (mean wheal diameter  = 2,9 mm, range 2–6 mm).

Fourteen out of the 28 patients with a positive PPT performed with fresh citrus fruit accepted to perform blood test: the mean values of sIgE against orange and the main pollens and plant derived foods are reported in Table 4; the geometric mean value of sIgE for orange was 3.5 kU/L (range: 0.85–39,2 kU/L). A direct correlation seems to exist between the high level of sIgE for the rPhl p12 and rPru p 3, homolog to the two orange allergens Cit s 2 and Cit s 3 respectively, and the generalized clinical symptoms (vomiting and urticaria, abdominal pain and diarrhoea) as reported in Table 3 for the patient 1 (sIgE  = 34.2 and 19.7 for rPhl p 12 and rPur p 3 respectively) and patient 3 (sIgE  = 4 and 16.6 for rPhl p 12 and rPur p 3 respectively).

**Table 4:** Pollinosis patients' clinical features.

ALLERGENS	n of positive/14 (≥ 0.35 kU/L)	g mean sIgE (kU/L)°	range (kU/L)°
*Orange*	9	3.5	0.85–39.2
*Phleum p.*	14	52.0	1.5–>100
*Betula v.*	13	8.2	0.4–>100
*Corylus a.*	14	7.0	0.4–92.7
*Olea e.*	13	7.5	0.8–70.8
*Parietaria j.*	13	5.2	0.36–43.2
*r Phl p 1*	12	49.2	15.1–>100
*r Phl p 12*	11	2	0.35–34.2
*r Bet v 2*	4	9.6	2.38–47
*r Pru p 3*	5	4.83	1.07–19.7
MuxF3-CCD	5	1.14	0.42–6.43
**CITRUS FRUITS**	**N of positive PPT (%)** §		**PPT **Mean wheal diameter mm (range) §
**Orange**	**24/72 (33)**		2.8 (2–7)
**Lemon**	**16/72 (22)**		2.8 (2–6)
**Clementine**	**12/72 (17)**		(2–6)

Levels of specific IgE (sIgE) against the main pollens (*Phleum p., Parietaria j., Olea e., Betula v., Corylus a*.), fruit extracts (orange, apple, peanut, wheat), recombinant allergens (rPhl p 1, rPhl p 12, rBet v 2, rPru p 3) and MuxF3-CCD in children and young adults with pollinosis. The geometric mean values of sIgE were calculated on the 14 out of the 28 patients with a positive reaction to prick-to-prick (PPT) with fresh citrus fruit pulp who accepted to perform blood test.

g mean: geometric mean; N: number; sIgE: specific IgE; PPT: prick-to-prick test.

§ Calculated on the 72 patients enrolled in the study.

° Calculated on the 14 patients who perform blood test.

### Expression analysis of sensitizing factors in citrus pollen

**Figure 1:**
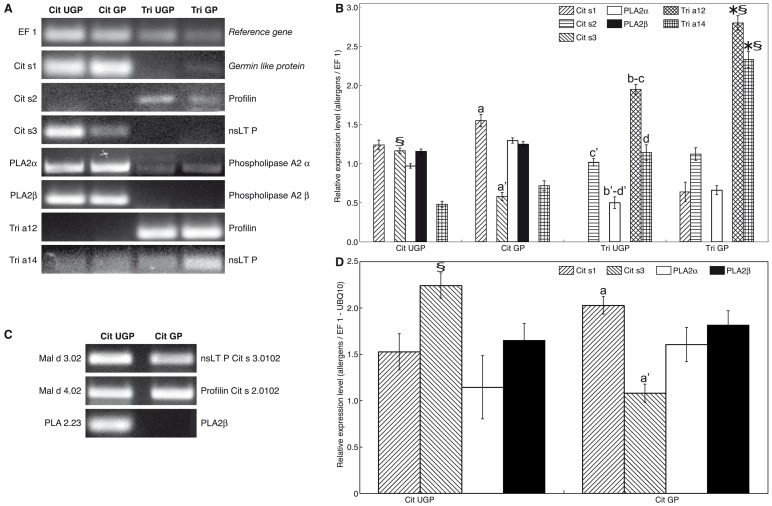
Allergens and phospholipase expression analysis in C*itrus clementine* and *Triticum aestivum* pollens. The cDNAs obtained from mRNA of *Citrus c*. (Cit) and *Triticum a*. (Tri) pollens ungerminated (UGP) and germinated (GP) have been tested for the different allergens (Cit s 1, Cit s 2, Cit s 3, Tri a 12 and Tri a 14) and phospholipase isoforms (PLA2 α and PLA2 β) through semi-quantitative RT-PCR after 40 cycles. EF-1 expression was used as reference gene (**A**). Lanes: *Citrus clementine* pollen ungerminated: Cit UGP; *Citrus clementine* pollen germinated: Cit GP; *Triticum aestivum* pollen ungerminated: Tri UGP; *Triticum aestivum* pollen germinated: Tri GP. Densitometries of the PCR bands revealed through semi-quantitative RT-PCR (**B**). Data are expressed as allergen/reference gene ratio ± SD. EF-1 expression was used as reference gene. The cDNAs of *Citrus clementine* UGP and GP have been tested for two different apple allergens (Mal d 3.02 and Mal d 4.02), and one isoform of apple secretory phospholipase (PLA 2.23) through semi-quantitative RT-PCR after 40 cycles (**C**). Logarithmic histogram (LOG10) of the expression levels of the different allergens (Cit s 1, Cit s 2, Cit s 3) and phospholipase isoforms (PLA2 α and PLA2 β) obtained through qRT-PCR in samples of *Citrus c*. pollen (Cit UGP and Cit GP) after 40 cycles of reaction (**D**). Data were calculated on the basis of the quantitative-comparative Ct (ΔΔCt) method normalized to the geometric mean of two housekeeping genes (EF-1 and UBQ 10), chosen according to the geNorm software. Values are the mean (n = 9) ± SD. Different letters (a–a', b–b', c–c', d–d') indicate means that are significantly different in allergens pairwise comparison (between bars marked by the same letter, i.e. a and a', b and b' etc.); * Statistical significance compared different allergens in the same sample (Tri GP); § Statistical significance compared the same allergen among the different samples (p≤0.05 with the Student's *t*-test [two-tailed distribution, two-sample equal variance]).

The gene expression analysis were conducted in order to study which allergens and other sensitizing factors were actually expressed even in the pollen, after the recently identification and characterization of the three major orange allergens in fruits [9], [10], [12], [15], [16]: transcripts for Germin-like proteins (GLP, Cit s 1), profilins (Cit s 2), non specific Lipid Transfer Proteins (nsLTP, Cit s 3) and two isoforms of secretory phospholipase A2 (PLA2 α and PLA2 β) were tested in ungerminated (UGP) and germinated (GP) pollen of *Citrus clementine* (Cit UGP and Cit GP) and *Triticum aestivum* (Tri UGP and Tri GP) through either semi-quantitative RT-PCR (Figure 1A) and qRT-PCR (Figure 1D). The evaluation of the expression profile of the *Cit s* 1–3 allergens by semi-quantitative RT-PCR (Figure 1A) allowed to verify the presence of Cit s 1 (GLP) and Cit s 3 (nsLTP) transcripts even in the citrus pollen with a different pattern of expression than those observed in the literature for the orange fruits [12]: for the citrus pollen the two major allergens are the Cit s 1 and Cit s 3, whereas the expression of Cit s 2 is not detectable according to the experimental conditions described in Material and Methods section. The densitometry analysis (Figure 1B) performed on the semi-quantitative RT-PCR shown in Figure 1A, confirmed differences in the expression profile between Cit UGP and Cit GP: Cit s 1 and Cit s 3 are both expressed almost with the same intensity in UGP, whereas in GP Cit s 1 showed an higher expression signal (about 2.5 fold) when compared to Cit s 3 (1,55±0,08 Cit s 1 *vs* 0,58±0,05 Cit s 3); as concerning PLA2 isoforms, both enzymes were expressed more in Cit GP (1,3±0,03 PLA2 α *vs* 1,25±0,03 PLA2 β) compared to Cit UGP (1±0,03 PLA2 α *vs* 1,16±0,03 PLA2 β).

**Figure 2:**
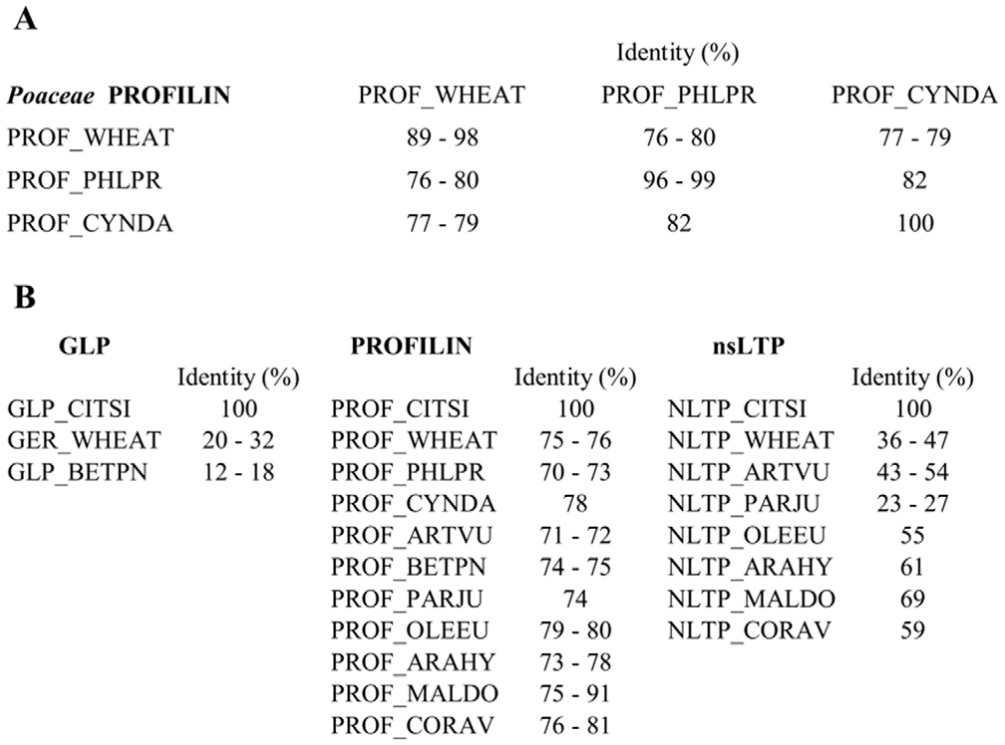
ClustalΩ multiple sequence alignments of allergen sequences annotated in UniProtKB database. Identity degree values expressed as multiple sequence alignment percentages of *Triticum aestivum* (PROF_WHEAT, Uniprot ID P49232-34, B6EF35), *Phleum pratense* (PROF_PHLPR, Uniprot ID P35079, O24650, O24282) and *Cynodon dactylon* (PROF_CYNDA, Uniprot ID O04725) profilin proteins (**A**). Identity percentages obtained from *Citrus sinensis* Cit s 1 (GLP), Cit s 2 (PROFILIN) and Cit s 3 (nsLTP) multiple sequence alignments (**B**). First column: *Citrus sinensis* GLP (GLP_CITSI, Uniprot ID P84159) with those for *Triticum aestivum* (GER_WHEAT, Uniprot ID P15290, B9VR55, C3UZE8, P26759, Q9SM34, Q9LD27, Q70PK0) and *Betula pendula* (GLP_BETPN, Uniprot ID P85352-54, P85336). Second column: *Citrus sinensis* PROFILIN (PROF_CITSI, Uniprot ID P84177) with those of *Triticum aestivum* (PROF_WHEAT, Uniprot ID P49232-34, B6EF35), *Phleum pratense* (PROF_PHLPR, Uniprot ID P35079, O24650, O24282), *Cynodon dactylon* (PROF_CYNDA, Uniprot ID O04725), *Artemisia vulgaris* (PROF_ARTVU, Uniprot ID Q8H2C8-9), *Betula pendula* (PROF_BETPN, Uniprot ID A4K9Z8, P25816), *Parietaria judaica* (PROF_PARJU, Uniprot ID Q9XG85, Q9T0M8), *Olea europaea* (PROF_OLEEU, Uniprot ID O24169-71), *Arachis hypogaea* (PROF_ARAHY, Uniprot ID Q9SQI9, Q5XXQ5, D3K177), *Malus domestica* (PROF_MALDO, Uniprot ID Q9XF40-42), *Corylus avellana* (PROF_CORAV, Uniprot ID A4KA40-45, Q9AXH4-5). Third column: *Citrus sinensis* nsLTP (NLTP_CITSI, Uniprot ID Q8L5S8, Q6EV47) with those of *Triticum aestivum* (NLTP_WHEAT, Uniprot ID Q5NE27-31, Q2PCB0, Q2PCB7-8, Q2PCD1-2, Q84N29), *Artemisia vulgaris* (NLTP_ARTVU, Uniprot ID P0C088, C4MGH0-2, C4MGG9), *Parietaria judaica* (NLTP_PARJU, Uniprot ID P43217, P55958, O04403-4, Q40905), *Olea europaea* (NLTP_OLEEU, Uniprot ID B2BGS3), *Arachis hypogaea* (NLTP_ARAHY, Uniprot ID B6CEX8, B6CG41), *Malus domestica* (NLTP_MALDO, Uniprot ID Q5GLH0, Q9M5X7, Q5J026), *Corylus avellana* (NLTP_CORAV, Uniprot ID Q9ATH2). Data are presented as range of percentage identity values as it has been considered all the allergen isoforms annotated and reviewed in UniProtKB database.

Since clinical data reported in literature [12] often shown that allergic patients to citrus fruits also manifest sensitization to grass pollens, expression analysis of *Cit s* 1–3 and grass specific allergens has been assessed to confirm cross-reactivity between pollens; among grass allergens have been chosen those from *Triticum aestivum* (wheat) pollen (Figure 1A and Figure 1B): Tri a 12.0103 (GenBank ID X89827.1), a profilin, and Tri a 14.0101 (GenBank ID AJ784902), a nsLTP. They represented conserved allergens among the most allergenic species of the *poaceae* family (i.e. *Triticum aestivum, Cynodon dactylon* and *Phelum pratense*) as shown by the multiple sequence alignments performed with ClustalΩ for profilin proteins, that shared identity values in the range between 76–82% (Figure 2A); on the contrary only isoforms for *Triticum a*. nsLTP proteins were annotated in the UniProtKB database, while sequences for *Cynodon d*. and *Phelum p*. were not yet present. Since one of the aims of the present study was to investigate on molecules involved in the cross-allergenicity citrus *vs* grass pollens, we have chosen that of *Triticum aestivum* as model.

The expression profiles performed through semi-quantitative RT-PCR (Figure 1A) and the densitometry analysis reported in Figure 1B showed that wheat allergens were remarkably expressed in *Triticum a*. pollen, with significantly differences between Tri UGP and Tri GP: GP showed the highest expression levels of both allergens with Tri a 12 more expressed than Tri a 14; the difference between Tri a 12 and Tri a 14 is higher in Tri UGP, where Tri a 12 showed an almost double fold expression level than Tri a 14. Moreover, we observed a faint signal for Cit s 1 and Cit s 2 allergens and PLA2 α enzyme also in wheat pollen grain (Figure 1A), with Cit s 1 expressed only in Tri GP and Cit s 2 and PLA2 α in both Tri UGP and Tri GP, emphasizing the cross-sensitization between citrus and grass pollens. Finally, it should be noted as the Tri a 14 is also expressed in *Citrus c*. pollen, both Cit UGP and Cit GP, emphasizing the pan-allergenic nature of nsLTPs.

Unexpectedly, the presence of allergens with common sequence has been observed also between *Citrus c*. and *Malus domestica* pollens. Among all the apple allergen sequences tested (known to be present also in apple fruits), it has been obtained signal for two specific allergen isoforms (Mal d 3.02, an nsLTP and Mal d 4.02, a profilin) and for potential sensitizing factors, PLA2 (isoform 23): in fact, these three pairs of primers were able to generate transcripts in *Citrus c*. both Cit UGP and Cit GP (Figure 1C) with different expression profile as Mal d 3.02 and PLA 2.23 showed an higher signal in UGP when compared to GP. These data were confirmed by the multiple sequence alignments for *Citrus c*. and *Malus d*. proteins, which shared values of 69% identity Mal d 3 *vs* Cit s 3, 90% identity Mal d 4 *vs* Cit s 2 and 77% identity PLA 2.23 *vs* PLA2 β.

To confirm previous results and to obtained quantitative data about allergens expression, the qRT-PCR methodology was followed and data are shown in Figure 1D. The technique of qRT-PCR combined with the use of highly specific primers designed for each allergen allowed to test in a precise, accurate and sensitive manner the gene expression at the level of messenger RNA. The selection of the optimal reference target has been performed by geNorm software [28]: in this experimental situation the optimal number of housekeeping genes was 2, represented by EF1 and UBQ10 (geNorm V <0.15 when comparing a normalization factor based on the 2 or 3 most stable targets). As shown in Figure 1D, Cit s 2 was not detected even after 40 cycles; Cit s 1 was higher expressed in Cit GP when compared to Cit UGP (2,03±0,02 in Cit GP *vs* 1,53±0,1 in Cit UGP), whereas Cit s 3 expression was higher in Cit UGP (2,24±0,1) than in Cit GP (1,08±0,14), being also the allergen with the highest level detected in *Citrus c*. pollen. As described before and confirmed also by qRT-PCR for Cit GP, Cit s 1 showed an expression signal double higher than Cit s 3. The two isoforms of PLA2 (PLA2 α and PLA2 β), were differentially expressed in Cit UGP and Cit GP with a stronger signal for PLA2 β in respect to PLA2 α, showing the same trend reported for the densitometry analysis (Figure 1B): an higher signal in Cit GP (1,61±0,34 PLA2 α *vs* 1,81±0,18 PLA2 β) when compared to Cit UGP (1,14±0,18 PLA2 α *vs* 1,65±0,15 PLA2 β) for both PLA enzyme isoforms.

According to the World Health Organization (WHO), two proteins can be considered cross-allergens if they share 35% identity on 80 residues and have an exact match of 6–8 residues on a single peptide (http://www.codexalimenatarius.net) [32]. On these basis and according to the data reported both in literature [12], [33], [34] and in the clinical results section about cross-allergenicity, we performed a bioinformatics approach based on the ClustalΩ multiple sequence alignments to find sequence homologies among the three main allergens in *Citrus sinensis* (Cit s 1, Cit s 2 and Cit s 3) and the same class of proteins, that may be present in the well known allergenic plant species considered in the clinical trials (i.e. *Triticum aestivum, Phleum pratense, Cynodon dactylon, Betula pendula, Artemisia vulgaris, Parietaria judaica, Malus domestica, Corylus avellana, Olea europaea, Arachis hypogaea*). As reported in Figure 2B, profilin proteins were the most conserved and with the highest identity percentage values (70–90%); on the contrary, nsLTP sequence identity values were lower in the range between 23% Cit s 3 *vs Parietaria judaica* to 69% Cit s 3 *vs Malus domestica*; as regards GLP, the identity values didn't exceed the percentages of 35% to consider them cross-allergens (it was in the range 12–32%).

## Discussion

Pollen allergens studies and sensitization patients' analysis to plant allergens describe a picture in which allergies to citrus fruit and cross-allergies with other pollen/fruit proteins could play an important role in the insurgence of sensitization and/or allergy reactions.

The allergy to citrus fruits is not clinically relevant as other foods and pollens (www.foodallergyitalia.org), even if some early studies suggested oranges being an important allergenic food [9], [10], [12], [35]. This is possibly due to the fact that a very high percentage of sensitization to citrus is not accompanied by clinical reactivity to these fruit, because only one third of self-reported reactions were confirmed by oral provocations [12]; it is also evident that clinical orange allergy exists and confirmed by challenge tests [10], [15].

As in literature few data are reported about allergy to citrus fruits, above all in paediatric population, the aim of this study was to investigate this topic in a population of children and young adults with pollinosis. Interestingly, we found a relevant percentage of sensitisation (39%) to citrus fruits in the population of pollen allergic patients recruited. Moreover, three out of the 72 pollen allergic patients (4%) presented adverse reactions after the ingestion of orange (2 patients) and clementine (1 patient) and in all of them the IgE- mediated mechanism has been confirmed by the positive response to the PPT with the fresh citrus fruit pulp. In a previous study, Kumar et al. [36] described a lower percentage (9.2%) of citrus fruit sensitization in 216 Indian children and adults affected from asthma. Asero et al. [37] reported that out of 200 children and adults with pollen allergy, 7 (3.5%) presented adverse reactions to a citrus fruit (orange, tangerine) with a similar percentage obtained also for the present study. Moreover, the present data reported that 2 out of 3 citrus allergic patients presented systemic reactions (urticaria, gastrointestinal symptoms) and 1 patient OAS after the ingestion of citrus fruits. The 2 patients with the generalized clinical symptoms showed high level of sIgE for the rPhl p12 and rPru p 3, homolog to the two orange allergens Cit s 2 and Cit s 3 respectively, emphasizing a direct correlation between sensitization/specific antibodies levels and the allergenic symptomatology. In particular, profilin are mainly involved in local symptoms (i.e. OAS) [15] contrary to systemic symptomatology mainly due to a sensitization towards nsLTPs, as highlighted by our clinical data.

The other patient with OAS has clear skin sensitization but not significant positivity to profilins and nsLTPs: probably other allergenic molecules are implied for such symptoms.

As suggested from previous works [38], [39], patients allergic to pollen may present different kinds of symptoms after eating several plant foods due to the cross-reactivity of pollen allergen specific IgE that reacts with homologous plant food proteins. Examples for such cross-reactive allergens are profilins as Phl p 12 or Bet v 2, which are recognised by IgE from 10% to 20% of pollen-allergic patients [40] and cause IgE cross-reactivity among botanically unrelated pollen and between pollen and food [41], [42]. In a previous study [15] about the evaluation of different pattern of allergen recognition in 6 orange allergic children, all the patients with isolated OAS presented sensitization to a profilin (Bet v 2), and the 2 patients with systemic reactions (general discomfort, urticaria) were Bet v 2 negative. In contrast, in the present study the patients recruited with generalized symptoms were all Bet v 2 positive and the one with OAS resulted Bet v 2 negative. Moreover all patients who presented a positive PPT reactions to citrus fruit, included the three patients with citrus fruit allergy, presented a positive response to SPT with grass pollen extract and/or the presence of sIgE against *Phleum p*., which belongs as *Triticum a*. to *poaceae* family. The presence of cross-reactivity between grass and citrus pollen has been described even by El-Qutob Lòpez et al. [43], who reported a case of systemic reaction (urticaria and angioedema) after ingestion of orange blossom pollen in a woman sensitized to several pollens included the grass one. In conclusion the present clinical data confirm a relevant prevalence of citrus fruit sensitization and show that orange and clementine allergy is not a so rare condition in pollen sensitized patients.

The molecular characterization of citrus pollen and cross-allergenicity was aimed to verify whether some important citrus allergen genes (Cit s 1, Cit s 2 and Cit s 3), known to be expressed in the fruit and to provoke allergic reactions after ingestion [12] were also expressed in pollen.

In the international allergen database (http://www.allergen.org/index.php) a single GLP, a single profilin and two nsLTPs were listed as allergens in sweet orange (*Citrus sinensis*). We demonstrated the expression of these allergens also in *Citrus clementine* pollen and in particular, Cit s 1 and Cit s 3 represent the two allergens commonly expressed in fruit and pollen, whereas Cit s 2 is present in citrus fruit and not detectable in citrus pollen. Hyun and Kim [44] reported the genomic identification of putative members of *Citrus c*. allergens, five different isoforms of nsLTP (Cit c 3) and three different isoforms of profilin (Cit c 2) annotated on Phytozome (http://www.phytozome.net/) and the Citrus Genome Database (http://www.citrusgenomedb.org/); the molecular data presented in this study demonstrated the presence of a GLP also in clementine pollen.

About profilin, when the RT-PCR was performed with the primers for a profilin isoform of *Malus domestica* fruit, the *Citrus c.* pollen showed a clear band either in Cit UGP and Cit GP; however, this result was not obtained when the RT-PCR was assessed with primers specific for the *Citrus sinensis* profilin. The data could mean that the isoform of *Citrus sinensis* annotated in UniProtKB database is not present in clementine pollen. Interestingly, the Cit s 2 isoform present in citrus fruit is also present in *Triticum a*. pollen, supporting the hypothesis of a cross-sensitization between *poaceae* pollen and citrus fruits. On the other hand, the Tri a 14, the nsLTP specific of *Triticum a*. is present in citrus pollen both Cit UGP and Cit GP, emphasizing the pan-allergenic nature of nsLTPs. As *Triticum a*. profilin and nsLTP protein sequences are annotated in UniProtKB database, we verified their homology sequences among the well known species of *poaceae* family considered allergenic (i.e. *Phleum pratense* and *Cynodon dactylon*) to assessed the degree of sequences identity and the possible cross-allergenicity between citrus and grass pollens. From the bioinformatics data, we showed that profilin were the most conserved proteins and with the highest identity percentage values, whereas nsLTP sequence identity values were lower; on the contrary, GLP cannot be considered involved in the cross-allergenicity, being the identity values lower than 35%.

Interestingly, we demonstrated both through clinical data and molecular approaches that *Citrus c*. and *Malus d*. specific allergen isoforms (Mal d 3.02, an nsLTP and Mal d 4.02, a profilin) shared high percentage identity values, confirming the possible cross-allergenicity citrus/apple.

The presence of phospholipase A2 (PLA2) in pollen of *Arabidopsis thaliana* has been reported only recently [45]; although the proven involvement of plant PLA2s in many biological functions, including i.e. senescence, wounding and stress responses, relatively little is known about plant PLA2s, and their genes essentially remain uncharacterized. In *Arabidopsis t*. three of four PLA2 paralogs (PLA2 β, γ and δ) have been characterized and found to be expressed in pollen, localized to the endoplasmic reticulum and/or Golgi, and playing critical roles in pollen development and tube growth.

A novelty of the present work is the finding of the PLA2 α and β in *Citrus c*. pollen, either UGP and GP, as in fruits, as well as the presence of PLA2 α also in *Triticum a*.; being PLA2 able to generate pro-inflammatory factors as eicosanoid substances, this enzyme could represent a key factor in the inflammatory response to allergens possibly activating the inflammatory cascade. From the apple genome database (http://genomics.research.iasma.it), we found that the apple PLA2.23 is conserved also in Cit UGP as shown by the semi-quantitative RT-PCR; the sequence alignments of PLA2.23 with PLA2 β from citrus fruit showed that the two sequences are very conserved [identities  = 116/150 (77%), positives  = 129/150 (86%)].

In conclusion, according to the molecular analysis performed in this work, Cit s 1 and Cit s 3, but also profilin, even if a different isoform from that expressed in orange fruit, and two isoforms of PLA2 enzyme (PLA2 α and PLA2 β) were identified as important allergens and sensitizing factors in *Citrus c*. pollen UGP and GP. As consequence, data obtained support the evidences that cross-sensitization among citrus and other plant homolog allergens, could play an important role in inflammatory response to citrus proteins and emphasize that citrus allergy can be considered a relevant food related allergy in pollen sensitizing patients.

Finally, we can hypothesize that allergy to oranges can occur mainly through local symptoms (i.e OAS and erythema) unlike other food allergy, such as apple or peach, characterized with more generalized symptoms such as vomiting, diarrhea and abdominal pain. The clinical data presented support the hypothesis that a sensitization towards nsLTPs mainly cause a systemic symptomatology and the relevant high percentages (39%) of children and young adults sensitized to citrus fruits recruited for our monocentric observational study suggest that there is a risk to develop an allergenic symptomatology in the future.
